# A Rare Case of Chylothorax after Heart Transplantation

**DOI:** 10.1155/2019/2049704

**Published:** 2019-10-21

**Authors:** Amit Alam, Linle Hou, Dreamy James, Kanika Mody, Deepa Iyer, Aziz Ghaly, Jesus Almendral

**Affiliations:** ^1^Division of Cardiology, Baylor University Medical Center, Dallas, TX, USA; ^2^Division of Cardiology, Rutgers-Robert Wood Johnson University Hospital, New Brunswick, NJ, USA; ^3^Division of Cardiology, Jersey Shore University Medical Center, Neptune, NJ, USA; ^4^Division of Cardiology, Hackensack University Medical Center, Hackensack, NJ, USA; ^5^Department of Cardiothoracic Surgery, Rutgers-Robert Wood Johnson University Hospital, New Brunswick, NJ, USA

## Abstract

Chylothorax is an exceedingly rare but serious complication of orthotopic heart transplantation (OHT). Prompt diagnosis and appropriate management are essential for a good outcome. Management is similar to that of nontransplant patients, but special attention must be given to patients' nutritional and immunological status. Relevant literature on this topic is limited. We describe our experience in the management of chylothorax after OHT and provide a summary of reported cases of this complication after isolated heart and combined heart/lung transplant.

## 1. Introduction

Chylothorax is an uncommon but serious complication following cardiothoracic surgery. It presents with unilateral or bilateral pleural effusion, dyspnea, and increased pleural drain output and is sometimes associated with life-threatening tamponade. Chylothorax complicating OHT is exceedingly rare and has only been described in medical literature in case reports and small case series [1–5]. Chyle leakage is a challenge in these immunosuppressed patients given the concern for infection and the requirements of rigorous dietary modification. Prompt diagnosis and timely treatment are of paramount importance.

## 2. Case

A 61-year-old female with end-stage ischemic cardiomyopathy on home milrinone listed as status 1B was admitted for heart transplantation. She had coronary bypass surgery 6 years prior and had a left-sided defibrillator implanted 4 years previously. The operation was uneventful, and the defibrillator lead and generator were explanted at the time of transplant. She was extubated on postoperative day (POD) 2 and was placed on standard immunosuppression medications and infection prophylaxis as per our center's protocol. On POD 5, the patient was noted to have excessive milky output from the left pleural drain that was placed intraoperatively. Fluid analysis showed lymphocytic predominance with pleural fluid triglyceride of 470 mg/dl and plasma triglyceride of 85 mg/dl confirming chylous drainage. Fluid staining was negative for bacteria, mycobacteria, and fungi. Management with low-fat diet and subcutaneous octreotide 100 mcg every 8 hours was initiated, and subsequently, *nil per os* (NPO) with total parenteral nutrition (TPN) was attempted to reduce chyle production. However, the patient continued to have persistently high output after 7 days (550 to 1,520 ml/day). Invasive intervention was discussed with the patient but she refused. The high output persisted despite conservative management until the patient finally agreed to an intervention. As she was deemed to be a high surgical risk due to posttransplant immunosuppression, she underwent interventional radiology-guided lymphangiography on POD 21 which demonstrated thoracic duct laceration at the level of the left clavicle that was successfully embolized. The pleural drain output decreased substantially and the chest tube was subsequently removed. The patient was discharged home on POD 25 with no recurrence.

## 3. Discussion

First described by Olof Rudbeck and Jean Pecquet in the 17^th^ century, the lymphatic system consists of the lymph glands, lymphatic vessels, cisterna chyli, and thoracic duct [6]. In the abdomen, the 4 main lymphatic trunks coalesce along the vertebral column at the level of L2 to form the cisterna chyli. From there, the lymph is transported to the chest via the thoracic duct which extends from L2 to the base of the neck. The duct is 2-5 mm in diameter and varies in length from 38 to 45 cm. It collects lymph from most of the body aside from the right side of the head and neck, right upper thorax, and right upper extremity which are drained by the right lymphatic duct. From its origin at the superior pole of the cisterna chyli, the thoracic duct traverses the aortic opening of the diaphragm between the aorta and azygous vein and ascends the posterior mediastinum to the right of the midline. At the T5 level, it gradually inclines to the left and ascends behind the aortic arch. In the neck, the thoracic duct forms an arch which rises 3-4 cm above the left clavicle and descends anterior to the first part of the left subclavian artery. It ends by the opening at the junction of the left subclavian and internal jugular veins [7].

The thoracic duct transports chyle and lymph from the gastrointestinal tract, abdominal wall, and lower extremities to the systemic venous system. Chyle contains large amounts of chylomicrons, triglycerides, fat-soluble vitamins, and cholesterol. Lymph, a constituent of chyle, contains significant amounts of immunoglobulins, lymphocytes, enzymes, and digestive products [8]. Chylothorax refers to injury to the thoracic duct as it transverses the thoracic cavity and the resulting leakage of chyle into the pleural space. The thoracic duct transports approximately 2.5 l of chyle a day, and any resulting injury could lead to the rapid accumulation of a large amount of fluid [9].

Postoperative chylothorax is a rare but serious complication with a reported incidence of 0.42% after general thoracic surgery [10]. It has been described following a broad range of surgical procedures with the highest rates (0.2-10.5%) reported following esophagectomy [11]. However, posttransplant chylothorax is exceedingly rare. An extensive literature search on MEDLINE and PubMed yielded only 7 reports of chylothorax after isolated heart or combined heart/lung transplant [1–5]. A summary of these reports, including the present case, is provided in Table 1. Four cases occurred after heart transplant and four after combined heart/lung transplantation. Patients were predominantly male (88%) with a mean age of 44 years (19-62 years old) presenting at a median of 9 days after surgery, mostly with a unilateral effusion. Three patients had prior cardiac surgery—one had an aneurysmectomy and endocardial resection, one had a left ventricular assist device implanted and replaced after 4 months, and one had prior coronary bypass surgery. Only one patient had acute rejection, and none developed an infection. Two cases required surgical reexploration via thoracotomy with suture ligation of the thoracic duct at the level of the carina in one case and ligation at the supradiaphragmatic level in the other. One had thoracic duct embolization (present case) while the rest resolved with conservative therapy. No patient had recurrence of chylothorax one year after treatment.

Chylous pleural effusion usually develops 1-2 weeks postoperatively and typically presents with dyspnea, chest discomfort, cough, or increased drainage if a chest tube is in place [8]. The effusion could be right-sided (52.7%), left-sided (25.7%), or bilateral (21.6%) depending on the location of the leak. Thoracic duct injury above the fifth thoracic vertebra causes a left-sided effusion while damage below this level leads to an effusion on the right side [12, 13].

Iatrogenic thoracic duct injury during thoracic surgery is the most common cause of traumatic chylothorax [8]. Significant embryological variation in thoracic duct anatomy is present in 35% of the population and is a key factor in inadvertent duct injuries [6, 13]. In addition, thoracic aortic reoperations and descending aortic repairs have been reported as risk factors for postoperative chylothorax likely due to the presence of adhesions and altered location of the thoracic duct after reoperation [14].

Chylothorax has also been reported with central venous cannulation of the neck vessels as well as with duct blockage from catheter-related venous thrombosis [13]. Lymphangiography, if performed, helps elucidate the site and mechanism of thoracic duct trauma. In our case, lymphangiography showed ethiodized oil extravasation at the level of the left clavicle. A central venogram performed at the same time showed occlusion of the left subclavian vein at the level of the thoracic outlet with nonvisualization of the ostium of the thoracic duct. As the patient did not have central venous catheterization in her left neck, we postulate that the thoracic duct injury and resulting subclavian vein thrombosis likely arose from the extraction of the defibrillator lead in her left upper chest at the time of heart transplant.

Diagnosing chylothorax can be sometimes elusive and a strong clinical suspicion is important. The classic milky fluid color present in our case is seen in less than half of patients. In a study by Maldonado et al., chylous effusion was described as milky in only 44% of patients, with the rest documented as serous (26%), serosanguinous (26%), or bloody (3%). In addition, although majority of the effusions are exudative, up to 14% may be transudative, most commonly in patients with cirrhosis [12]. Diagnosis typically requires fluid analysis of pleural fluid with a triglyceride level of >110 mg/dl having a 99% chance of being chylous while fluid with a triglyceride level of <50 mg/dl has a likelihood of only 5% [15]. For fluid with triglyceride levels of <110 mg/dl but >50 mg/dl, lipoprotein electrophoresis demonstrating chylomicrons is required to confirm or exclude the diagnosis, although this test may not be widely available [8].

It is important to recognize chylothorax after transplant as prompt diagnosis and a multidisciplinary approach are necessary for a favorable outcome. Untreated postoperative chylothorax is associated with mortality rates of 50-82% [11]. Prolonged chyle loss could lead to dehydration and malnutrition, as well as hyponatremia, acidosis, and hypocalcemia [8]. Patients are also at increased risk of infections due to the loss of lymphocytes and immunoglobulins with resulting depression of both cellular and humoral immunity. Development of chylothorax is associated with a higher 30-day risk of sepsis, pneumonia, reintubation, reoperation, and death [16]. This is particularly problematic in posttransplant patients who are already immunosuppressed. Furthermore, prolonged lymphocyte losses could lead to a decrease in total lymphocytic count. This could lead to profound immune suppression particularly if lymphocyte-depleting antirejection medication is utilized posttransplant. In addition, cyclosporine, a common immunosuppression agent, is secreted in chyle [17]. In patients with prolonged and uncontrolled chyle loss, close drug level monitoring with appropriate dose adjustment is warranted.

Various treatment modalities for chylothorax, ranging from conservative to invasive interventions, have been described. Low-output chylothoraces (<1 l chyle/day) usually respond to conservative measures while high-output effusions (>1 l/day), commonly seen in postsurgical patients, typically need more invasive intervention [10]. With appropriate and timely intervention, mortality rates have decreased to 10-16% [11]. Management largely depends on experience from case reports and single-center case series as no comparative clinical trials of specific therapies are available.

The initial treatment of chylothorax involves thoracic cavity decompression via a chest tube (if one is not already in place) to relieve dyspnea and to quantitate daily chyle output to monitor response to treatment. Conservative management with dietary modification using low-fat diet and medium-chain fatty acids (MCFA) is usually attempted first. The MCFA diet is utilized because these fatty acids are absorbed directly into the portal venous system and bypass the lymphatic system including the thoracic duct. If this fails, temporary NPO with total parenteral nutrition to reduce chyle formation has been successful in some cases [8, 13].

Adjunctive therapy with somatostatin or its analogue octreotide, which was used in our case, is often utilized in addition to dietary modification. Somatostatin acts directly on vascular somatostatin receptors in order to minimize lymphatic fluid excretion. It also reduces lymphatic flow by increasing the splanchnic arteriolar resistance and decreasing gastrointestinal blood flow [6]. However, the dose, route of administration (subcutaneous, intravenous, or continuous infusion), or duration of treatment has not been established in prospective studies [18].

Conservative measures are aimed at reducing the formation and flow of chyle through the thoracic duct to allow the leak to close and eventually heal. It has a reported success rate of 16-75% [18]. However, surgical intervention should be strongly considered if the chyle output remains >1,000 ml/day (or > 10 ml/kg/day) for >5 days, chyle flow persists for >2 weeks, or if clinical signs of malnutrition or metabolic derangements occur [8, 10, 13, 16].

Operative treatment options include thoracic duct ligation and thoracic duct embolization or disruption. Thoracic duct ligation is traditionally performed via a thoracotomy. In a unilateral effusion, thoracotomy is made on the side of the effusion as the leak is likely located on that side. For bilateral chylothoraces, it is usually best to approach the right side. Given the morbidity and pain associated with thoracotomy, use of video-assisted thoracic surgery (VATS) has become an attractive alternative [19]. Regardless of the approach (thoracotomy or VATS), identification of the site of leakage is usually attempted preoperatively via lymphangiography or by enteral administration of cream or oil [9, 11]. Clips are then applied proximal and distal to the site of injury. If the leak cannot be easily identified, mass ligation of lymphatic tissue in the supradiaphragmatic region between the descending thoracic aorta and azygos vein should be performed [6]. In addition, fibrin glue is sometimes applied to the area [11]. With thoracic duct ligation, success rates of over 90% have been reported [6, 10].

For patients at high risk of reoperation, such as in our case, thoracic duct embolization or disruption is a viable alternative with a success rate of 46-74% [20]. It is a technically challenging procedure requiring lymphangiography via a pedal or intranodal approach to visualize the location of the leak. Cannulation of the thoracic duct can be difficult and is successful in about two-thirds of cases. In patients that can be cannulated, embolization with endovascular coils and/or liquid embolic agents resolves the chyle leak in 90% [21]. In cases where lymphatic catheterization is unsuccessful, duct disruption can be attempted using a 21- or 22-gauge needle to create holes in the thoracic duct to slow down lymphatic flow allowing for healing of the leak. Success rates with needle disruption range from 44 to 72%. Complication rates of 0% to 10.5% have been reported for thoracic duct embolization or disruption including embolization of the pulmonary artery and injection site infections after lymphangiography. Chronic diarrhea and leg and abdominal swelling have been reported as delayed complications [20].

For patients with refractory chylothorax, a pleuroperitoneal shunt may be effective with a success rate of approximately 80% [9]. It involves the creation of a subcutaneous or external connection between the pleura and peritoneum connected to a pump activated by light pressure [13]. The shunt can be removed once the chyle leak resolves.

In summary, we report a rare case of chylothorax following heart transplantation. Unexplained pleural effusion, either unilateral or bilateral, should raise suspicion even in the absence of the classic milky appearance of the pleural fluid. Elevated pleural fluid triglyceride level is a typical finding. Conservative management with chest tube placement and diet modification should first be attempted in conjunction with octreotide. Patients should be closely monitored for malnutrition, dehydration, and infections with adjustments made to immunosuppressive therapy accordingly. Lymphangiography may help identify the site and mechanism of thoracic duct injury. When conservative management fails, an invasive procedure to ligate the thoracic duct via thoracotomy, VATS, or IR-guided embolization should be considered early.

## Figures and Tables

**Table 1:** 

Reference	Age (yrs)	Gender	Transplanted organ	Days after transplant	Laterality	Prior cardiac surgery	Treatment	Outcome
Bowerman et al. [1]	62	Male	Heart	7	Left	Aneurysmectomy Endocardial resection	Chest tube NPO/feeding tube MCT	No recurrence Low lymphocyte count 300/mm^3^
Twomey [2]	32	Male	Heart	18	Right	None	Chest tube NPO/TPN	No recurrence Acute rejection
Shitrit et al. [3]	53	Male	Heart/lung	120	Bilateral	None	Chest tube Oral low-fat diet	No recurrence No infection/rejection
Ziedalski et al. [4]	50	Male	Heart/lung	35	Bilateral	None	Chest tube Oral MCT IV octreotide Oral aminocaproic acid	No recurrence No infection/rejection
	27	Male	Heart/lung	7	Left	None	Chest tube NPO Left thoracotomy Duct ligation at the level of carina	No recurrence No infection/rejection
	19	Male	Heart/lung	10	Right	None	Chest tube NPO/TPN VATS pleurodesis Right thoracotomy Supradiaphragmatic duct ligation	No recurrence No infection/rejection
Berdy et al. [5]	47	Male	Heart	8	Mediastinal	Left ventricular assist device implant; device exchange after 4 mos	Mediastinal tube Oral low-fat diet	No recurrence No infection/rejection
Present report	61	Female	Heart	5	Left	Coronary artery bypass grafting	Chest tube Oral low-fat diet SC octreotide NPO/TPN Thoracic duct embolization	No recurrence No infection/rejection

## Abbreviations

MCFA: Medium-chain fatty acids
NPO: Nil per os
OHT: Orthotopic heart transplant
POD: Postoperative day
TPN: Total parenteral nutrition
VATS: Video-assisted thoracoscopic surgery.
